# Cytotoxic Effect of Poly-Dispersed Single Walled Carbon Nanotubes on Erythrocytes *In Vitro* and *In Vivo*

**DOI:** 10.1371/journal.pone.0022032

**Published:** 2011-07-19

**Authors:** Sumedha Sachar, Rajiv K. Saxena

**Affiliations:** School of Life Sciences, Jawaharlal Nehru University, New Delhi, India; IIT Research Institute, United States of America

## Abstract

Single wall Carbon Nanotubes (SWCNTs) are hydrophobic and do not disperse in aqueous solvents. Acid functionalization of SWCNTs results in attachment of carboxy and sulfonate groups to carbon atoms and the resulting acid functionalized product (AF-SWCNTs) is negatively charged and disperses easily in water and buffers. In the present study, effect of AF-SWCNTs on blood erythrocytes was examined. Incubation of mouse erythrocytes with AF-SWCNTs and not with control SWCNTs, resulted in a dose and time dependent lysis of erythrocyte. Using fluorescence tagged AF-SWCNTs, binding of AF-SWCNTs with erythrocytes could be demonstrated. Confocal microscopy results indicated that AF-SWCNTs could enter the erythrocytes. Treatment with AF-SWCNTs resulted in exposure of hydrophobic patches on erythrocyte membrane that is indicative of membrane damage. A time and dose dependent increase in externalization of phosphatidylserine on erythrocyte membrane bilayer was also found. Administration of AF-SWCNTs through intravenous route resulted in a transient anemia as seen by a sharp decline in blood erythrocyte count accompanied with a significant drop in blood haemoglobin level. Administration of AF-SWCNTs through intratracheal administration also showed significant decline in RBC count while administration through other routes (gavage and intra-peritoneal) was not effective. By using a recently developed technique of a two step *in vivo* biotinylation of erythrocytes that enables simultaneous enumeration of young (age <10 days) and old (age>40 days) erythrocytes in mouse blood, it was found that the *in vivo* toxic effect of AF-SWCNTs was more pronounced on older subpopulation of erythrocytes. Subpopulation of old erythrocytes fell after treatment with AF-SWCNTs but recovered by third day after the intravenous administration of AF-SWCNTs. Taken together our results indicate that treatment with AF-SWCNTs results in acute membrane damage and eventual lysis of erythrocytes. Intravenous administration of AF-SWCNTs resulted in a transient anemia in which older erythrocytes are preferably lysed.

## Introduction

Nano-sized particles are finding increasing practical applications and commercial use. Consequently, the possibility of occupational exposure to nano-particles has increased. Furthermore, as the use of nanoparticles increases, they may diffuse and accumulate in environment increasing the risk of environmental exposure. Health effects of exposure to nanoparticles are now under investigation by many researchers [1], [2], [3]. Nanoparticles have a tendency to agglomerate into particles of larger size that may not have the same biological effects as non-agglomerated nanoparticles. For studying the biological effects therefore it is necessary to test non-agglomerated forms of nanoparticles. For Single walled Carbon Nanotubes (SWCNTs) disaggregation can be achieved by acid functionalization that introduces negative charge, without changing the essential structural features of SWCNTs [4], [5]. We have previously reported cytostatic and toxic effects of acid functionalized SWCNTs (AF-SWCNTs) on LA4 lung epithelial cell line [5]. We also found that the administration of AF-SWCNTs intratracheally resulted in cardiac damage in mice [6].

Since AF-SWCNTs were toxic to some types of nucleated cells that are capable of self repair, we hypothesized that the AF-SWCNTs may have a more aggravated toxic effect on erythrocytes that have no nucleus and lack substantial cellular repair mechanisms. This proposition was examined in the present study. Our results indicate that unlike pristine SWCNTs, AF-SWCNTs are significantly toxic to mouse blood derived erythrocytes *in vitro* as well as *in vivo*. AF-SWCNTs were found to bind with erythrocytes and cause significant membrane damage as well as externalization of phosphatidylserine. Administration of AF-SWCNTs intravenously induced a significant though transient anemia in mice. Older as compared to the younger subpopulation of erythrocyte in blood circulation, was more susceptible to the toxic effect of AF-SWCNTs. Taken together our results show that non-agglomerated form of SWCNTs may exert significant and rapid toxic effects on blood erythrocytes.

## Materials and Methods

### Experimental model

Inbred Swiss and C57BL/6 female mice (6–12 weeks old, 20–25 g body weight) were used throughout this study. Animals were bred and maintained in the animal house facility at JNU, New Delhi or obtained from the National Institute of Nutrition, Hyderabad. The animals were housed in positive-pressure air conditioned units (25°C, 50% relative humidity) and kept on a 12 h light/dark cycle. Water and mouse chow were provided *ad libidum.* All the experimental protocols were approved by JNU Institutional Animal Ethics Committee (IAEC Project Code: 5/2010) and performed accordingly.

### Cells and Reagents

For in vitro studies, erythrocytes derived from mouse blood were suspended in RPMI complete medium (RPMI-CM) containing glutamine (2 mM), HEPES buffer pH 7.2 (25 mM), gentamycin (20 µg/ml) obtained from Sigma-Aldrich (India) and fetal bovine serum, obtained from Hyclone (South Logan, UT). ANS (8-Anilino-1-naphthalenesulfonic acid) was obtained from Sigma-Aldrich (India). Alexa fluor 488/633 hydrazide was obtained from Molecular Probes (Carlsbad, CA). Biotin-X-NHS Ester (BXN) was from Calbiochem (La Jolla, CA) and Streptavidin Allophycocyanin (SAv-APC) and Annexin-V PE were obtained from BD biosciences (San Diego, CA). Single-wall carbon nanotubes (SWCNT's) were purchased from Sigma (Catalogue #: 636797, amorphous carbon <3%). Similar results were obtained for SWCNTs preparations procured from [Carbon Nanotubes Inc. Houston, TX, USA (purified HIPCO SWCNTs <15% ash)] and their derivatives.

### Acid functionalization of particles

Acid functionalized SWCNTs (AF-SWCNTs) were produced by suspending 20 mg of powder SWCNTs in 20 ml of 1∶1 concentrated HNO_3_:H_2_SO_4_ in 100 ml high-pressure vessels in a microwave digester as described previously [5]. Briefly microwave power was applied at 50% of 900 watt total and the pressure was controlled at 20±2 psi for 3 min resulting in an internal temperature of 138–150°C. Suspensions were cooled, diluted five-fold with H_2_O and dialyzed four times against 5 liter water over a two day period. Dialyzed suspensions were freeze dried, weighed and resuspended in 5 ml water. Particle size distribution and surface charge on AF-SWCNTs were as reported before [5]. The particle preparations were sonicated for 1 min in ice using a sonicator (Branson sonifier, VWR Scientific) prior to use.

### Attachment of fluorescent probes to AF-SWCNT

For covalent attachment of fluorescent probes to the nanotube surface, -COOH groups on AF-SWCNTs were exploited. AF-SWCNTs were suspended in water and treated with 1-ethyl 3-(3-dimethyl aminopropyl) carbodiimide (EDAC) and N-hydroxy succinimide (NHS) in order to get a succinimidyl intermediate. The mixture was continually shaken for 2 hrs and dialysed thereafter to remove excess NHS, EDAC and urea by-product. AF-SWCNTs thus activated were incubated with Alexa Fluor 488/633 hydrazide (Molecular Probes, Carlsbad, Ca) in dark with continuous mixing, followed by dialysis to remove free dye [7]. Attachment of fluorescence tag to AF-SWCNTs was confirmed flow cytometry.

### Confocal Microscopy of erythrocytes

Attachment or internalisation of AF-SWCNTs was confirmed by incubating erythrocytes with alexa fluor 488/633 tagged AF-SWCNTs, as described previously [7]. Erythrocytes were incubated in RPMI+1% Fetal Bovine Serum with fluorescenated AF-SWCNTs for 1 h and unbound/loosely bound particles were removed by three extensive washes with PBS, after that suspensions were analysed by Confocal Laser Scanning Microscope (Olympus Fluoview™ - FV1000).

### Measurement of membrane damage

Hydrophobicity of the erythrocytes was determined by staining with 8-anilino-1-naphthalenesulfonic acid (ANS) as described before [8]. Erythrocytes suspended in PBS were incubated with ANS (4 mM) at 37°C for 20 min. The binding of ANS to hydrophobic sites on erythrocyte membrane was measured (Ex_max_ = 365 nm) using a Fluorimeter (Spectramax M2^e^). Fluorescence spectra were scanned from 400 to 600 nm. The excitation and emission band passes were 5.0 nm in width.

### Particle Exposure

Erythrocytes were suspended in RPMI 1640 supplemented with 10% Fetal bovine serum and incubated with control and AF-SWCNT's at different concentrations over rotisserie (17 RPM) at 37°C for different time intervals. For *in vivo* studies, mice were given 100 µg AF-SWCNTs or saline control through intravenous, intraperitoneal, gavage and intratracheal routes and blood samples were taken at several time points thereafter. Blood erythrocyte count was determined by automated hematology analyser (Melet Schloesing Laboratories MS4e, Osny, France).

### *In vivo* biotin labelling

*In vivo* biotinylation of circulating erythrocytes was done as described previously [9], [10], [11], [12]. All blood erythrocytes were biotinylated by three daily intravenous injections of 1 mg of biotin-X-NHS Ester (BXN) dissolved in 20 µl of dimethylformamide (DMF) and 250 µl of phosphate buffered saline (PBS). Thirty days later, a second lower dose of B-X-NHS (0.6 mg) labelled all erythrocytes generated over thirty days with low intensity biotin. Blood erythrocytes derived from these mice 10 days after the second biotinylation step were used to enumerate old (biotin^high^ population, age>40 days), intermediate (biotin^low^, age 10 to 40 days), and young (biotin^negative^, age <10 days) populations of erythrocytes. Biotin high, low and negative erythrocyte populations could be identified and enumerated by staining with streptavidin-APC followed by flow cytometry as described before [9].

### Flow cytometry

To enumerate erythrocytes with high, low or no biotinylation, 1×10^6^ erythrocytes were stained with streptavidin allophycocyanin (APC) as described before [9]. For PS externalisation studies, erythrocytes were stained with annexin V-PE, by the procedure recommended by the manufacturer (BD Biosciences). Briefly, erythrocytes were suspended in annexin-V binding buffer (10 mM Hepes, pH 7.4, 140 mM NaCl, 2.5 mM CaCl_2_) and annexin-V PE for 20 min at 25°C in dark. Stained erythrocytes were immediately analysed on FACS Calibre flow cytometer (Becton Dickinson, San Jose, CA, USA) using Cell Quest software for acquisition and analysis. A minimum of 10,000 events were recorded for each sample.

### Statistical analysis

Each experiment was repeated at least three times. Statistical analysis by two-way ANOVA and student's t-test were done using Sigma plot and Sigma stat software. Data are presented as means ± SEM.

## Results

### Effect of control and acid functionalized SWCNTs on the recovery of murine erythrocytes in culture

**Figure 1:**
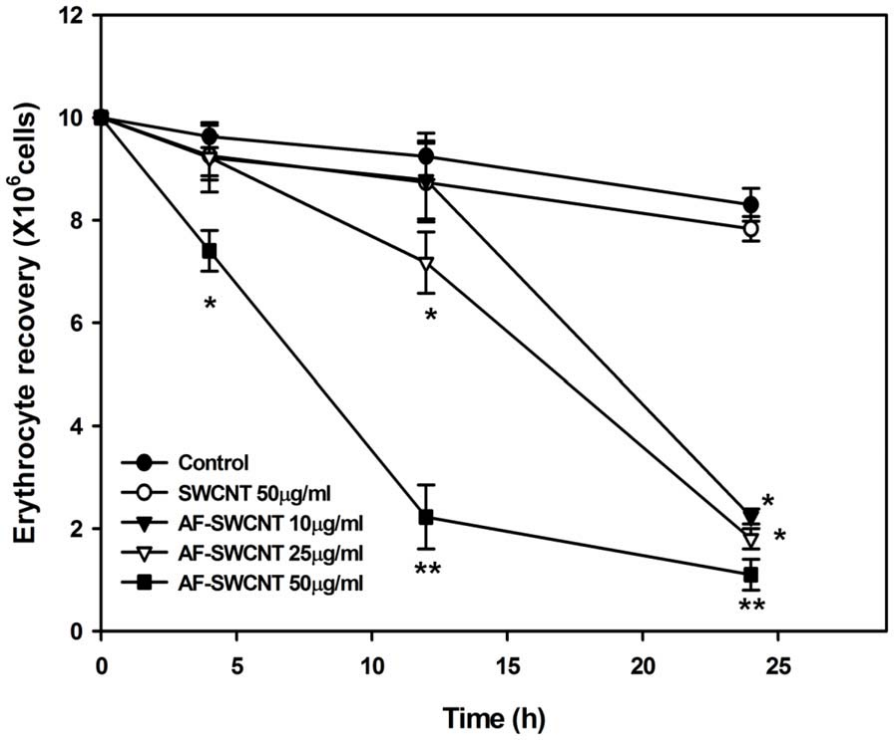
Effect of Control and AF-SWCNT's on erythrocytes *in vitro*. Erythrocytes isolated from Swiss mice were cultured in RPMI+10%FBS with different concentrations of control and AF- SWCNT preparations. After different time intervals, residual erythrocyte count was determined. Each point represents Mean±SEM. *p<0.01, **p<0.001 as compared to control untreated groups.

The effect of control and acid-functionalized SWCNTs was examined on murine erythrocytes. For this purpose, blood erythrocytes derived from female Swiss or C57BL/6 mice were cultured with control and acid-functionalized SWCNTs. To facilitate the interactions between carbon nanotubes and erythrocytes, the culture tubes were gently rotated (17 RPM) throughout the culture duration. Erythrocyte recoveries at different time points are shown in Figure 1. Treatment with control SWCNTs had no significant effect on the recovery of erythrocytes. A dose and time dependent decline (70 to 90%) in erythrocyte recovery was however seen in cultures treated with AF-SWCNTs. At 50 µg/ml concentration, a significant decline in erythrocyte recovery was seen even at the earliest 4 h time point. At the 24 h time point, erythrocyte recovery in AF-SWCNT treated cultures fell by 80-90%.

### Uptake of AF-SWCNTs by erythrocytes

**Figure 2:**
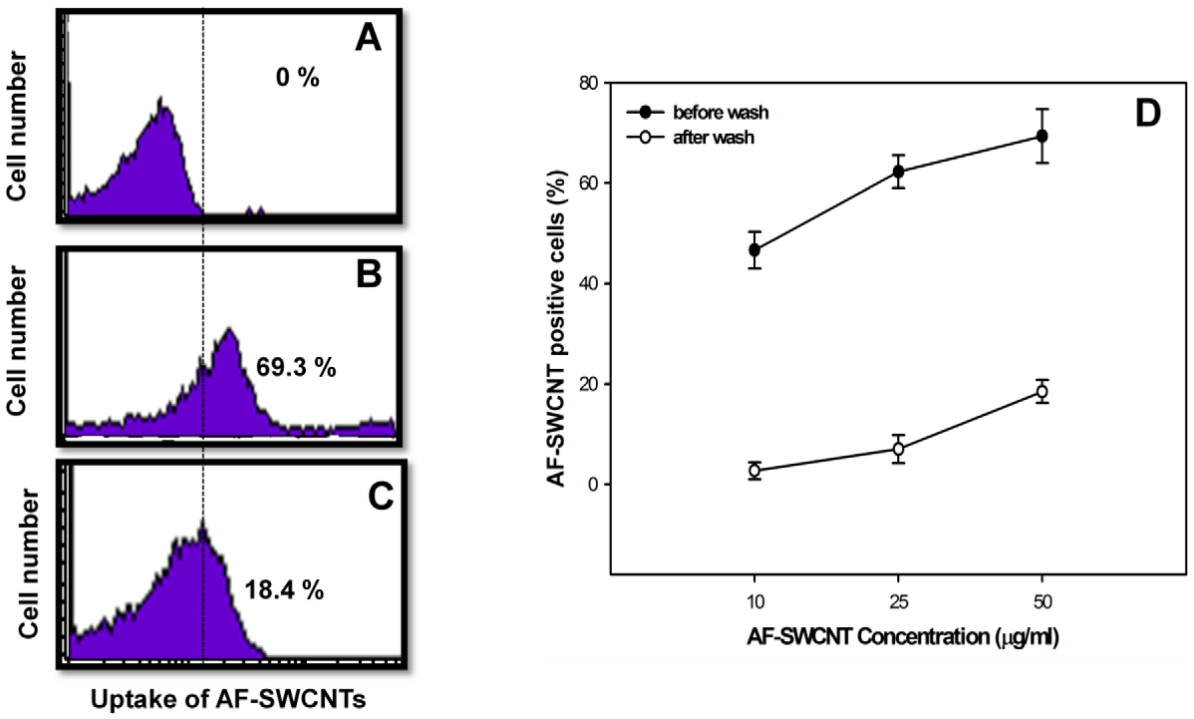
Uptake of fluorescenated AF-SWCNTs by erythrocytes *in vitro*. Erythrocytes were incubated with fluorescenated AF-SWCNTs (50 µg/ml) for 1 h and examined flow cytometrically either before (panel B) or after (panel C) three washings with fresh medium. Panel A shows the flow histogram for control unstained erythrocytes. Panel D shows the dose dependent uptake of AF-SWCNTs by erythrocytes with or without three washings. Results of a representative experiment have been shown. In panel D all data points represent mean ± SEM of three observations.

**Figure 3:**
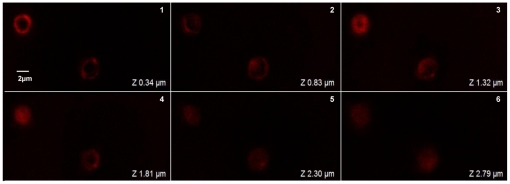
Localization of AF-SWCNTs in side erythrocytes by confocal microscopy. Fluorescenated AF-SWCNTs were prepared as described in Materials and Methods. Erythrocytes were incubated in RPMI +1%FBS with 50 µg/ml fluorescenated AF-SWCNTs for 1 h and unbound/loosely attached particles were removed by three extensive washings with PBS. After that erythrocyte suspension was taken for confocal microscopy. Above figure shows fluorescence images of erythrocytes incubated with alexa fluor 633 hydrazide tagged AF-SWCNTs as z-sections. (Magnification 100X).

For examining the interactions between AF-SWCNTs and erythrocytes, AF-SWCNT preparations tagged with fluorescence probe were used. Erythrocytes freshly derived from female C57BL/6 mice were incubated with fluorescence tagged AF-SWCNT particles at 50 µg/ml *in vitro* for 1 h and analysed on flow cytometer before and after washing the erythrocyte preparations. Results in Figure 2 (panel B) show that 69% of erythrocytes incubated with fluorescence tagged AF-SWCNTs were positive for fluorescence indicating an uptake or association with AF-SWCNTs. After washings 18.40% of the erythrocytes (panel C) were still positive for uptake of tagged AF-SWCNTs. These results suggest that AF-SWCNTs could associate with erythrocytes in a loose as well as a relatively stronger manner. Erythrocytes incubated with fluorescence tagged AF-SWCNTs were also examined by confocal microscopy. Results in Figure 3 show the localization of fluorescence in z-sections of erythrocytes and strongly suggest that some fluorescence tagged AF-SWCNTs could enter erythrocytes.

### AF-SWCNT induced membrane effects in erythrocytes

**Figure 4:**
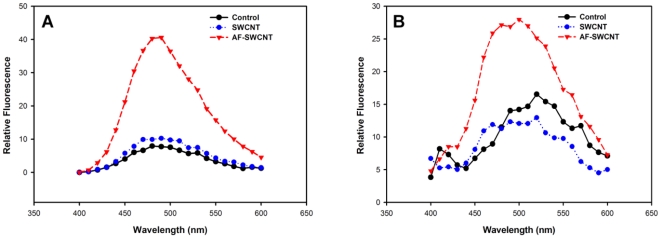
Effect of treatment with control and acid functionalized SWCNTs on hydrophobicity of erythrocyte membrane. Erythrocytes (5×10^6^ /ml in PBS+1%FBS at 37°C) from Swiss (panel A) and C57Bl6 (panel B) mice were incubated with 25 µg/ml of SWCNTs and AF-SWCNTs for 1 h. Cells were washed and incubated in PBS with 4 mM ANS for 20 min in dark at 37°C, after which fluorescence spectra was determined on a fluorimeter.

Exposure of hydrophobic regions in erythrocyte membrane is associated with cell damage [8]. In order to see if membrane hydrophobicity is induced in erythrocyte membrane exposed to AF-SWCNTs, freshly isolated mature erythrocytes were incubated with AF-SWCNTs at 37°C for 1 h and examined for membrane changes by using ANS (8-anilino naphthalene sulfonic acid), a dye that binds to hydrophobic patches on the cell membrane [13], [14]. Results in Figure 4 show that there was a significant increase in the binding of ANS to erythrocytes treated with AF-SWCNTs. A significant shift of emission maxima (blue shift) from 520 to 480 nm was also noted in AF-SWCNT treated erythrocytes. These results indicate a significant damage of erythrocyte membrane upon exposure to AF-SWCNTs.

**Figure 5:**
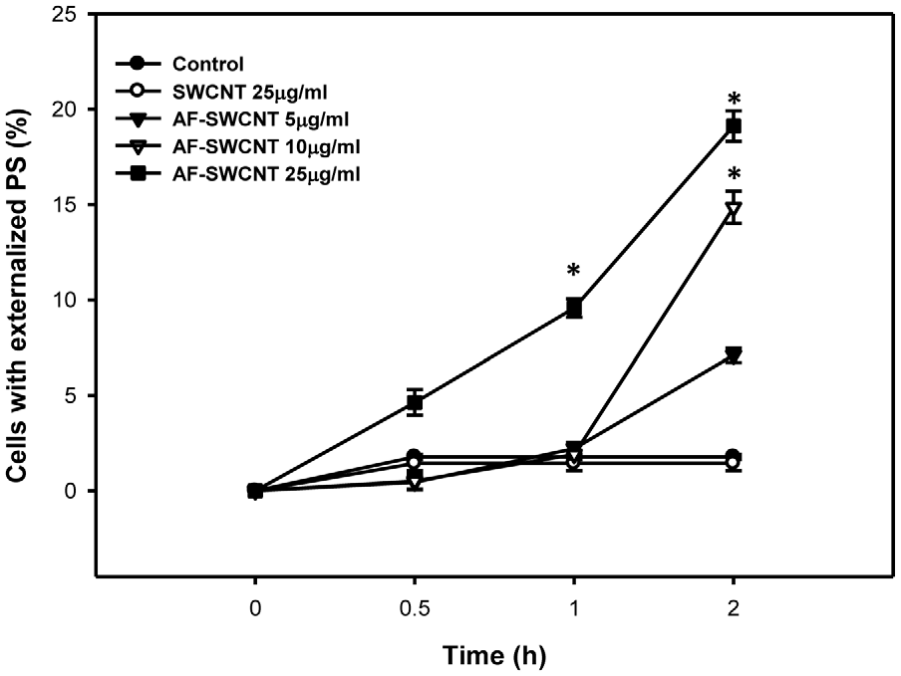
Effect of Control and acid functionalized SWCNTs on PS externalization in murine erythrocytes. Erythrocytes (0.5×10^6^ in 100 µl RPMI+1%FBS) from 6-8 weeks old C57BL/6 mice were incubated with given concentrations of control and acid functionalized SWCNTs for different time intervals at 37°C. PS externalization was studied staining with annexin V-PE antibody. Percent annexin V positive erythrocytes (mean ± SEM of three values) have been shown. *p<0.05 as compared to control.

Phosphatidylserine (PS) externalisation on cell membrane is an early marker of apoptosis and cell death [15]. Results in Figure 5 show a dose and time dependent increase in externalization of PS on erythrocytes incubated with AF-SWNCTs whereas no similar effect was seen upon incubation with control SWCNT preparation. Two hours after incubation with AF-SWCNTs, about 19% of the erythrocytes had externalized PS whereas in control erythrocytes as well as the erythrocytes treated with control SWCNTs, less than 2% cells expressed PS.

### *In vivo* effect of control and acid functionalized SWCNTs on blood erythrocytes

**Figure 6:**
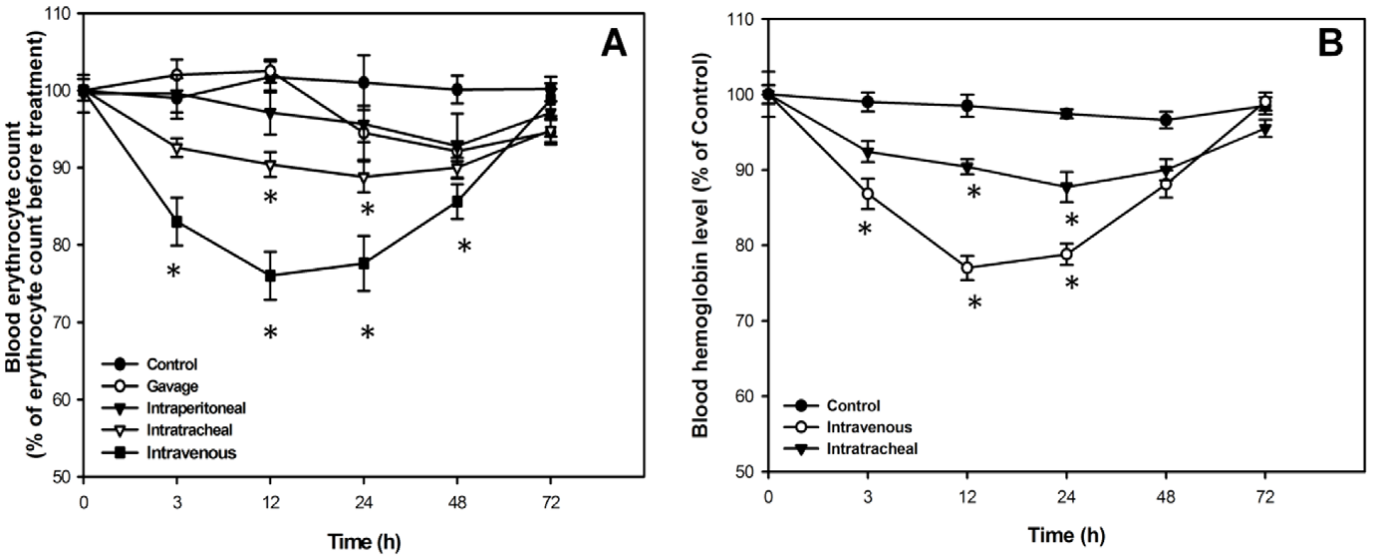
Effect of AF-SWCNT's on blood count of erythrocytes in mice. AF-SWCNT's were administered to 6–8 weeks old Swiss mice by the indicated routes (panel A). Mice were administered vehicle (PBS alone) or AF-SWCNT's (100 µg) intravenously, intratracheally, intraperitoneally, or the gavage route, and erythrocyte count was determined at different time intervals. Panel B shows effect of intravenously administered control and acid functionalised SWCNTs on hemoglobin content in blood. Results of a representative experiment have been shown. All data points represent mean ± SEM of three observations. *p<0.01 as compared to PBS administered control groups.

In order to determine if AF-SWCNTs exerted toxic effect on erythrocytes *in vivo* too, mice were administered control or acid functionalized SWCNTs through different routes and the erythrocyte count in blood was monitored. Results in Figure 6 (panel A) show that a single dose of AF-SWCNTs (100 µg) given intravenously resulted in a marked fall in erythrocyte blood count. Maximum decline in blood count was about 23% and occurred 12 and 24 h after the infusion of AF-SWCNTs. The blood erythrocyte count returned to normal levels 72 h after the administration of AF-SWCNTs. A lesser though significant fall in blood erythrocyte count (about 10%) was also observed when AF-SWCNTs were administered intratracheally (Figure 6A). Administration of AF-SWCNTs through oral gavage and intraperitoneal route did not result in a significant reduction in the blood erythrocyte count (Figure 6A). A concomitant transient drop in blood haemoglobin levels was also seen in mice administered a single dose of 100 µg of AF-SWCNTs through intravenous or intra tracheal route (Figure 6B).

**Figure 7:**
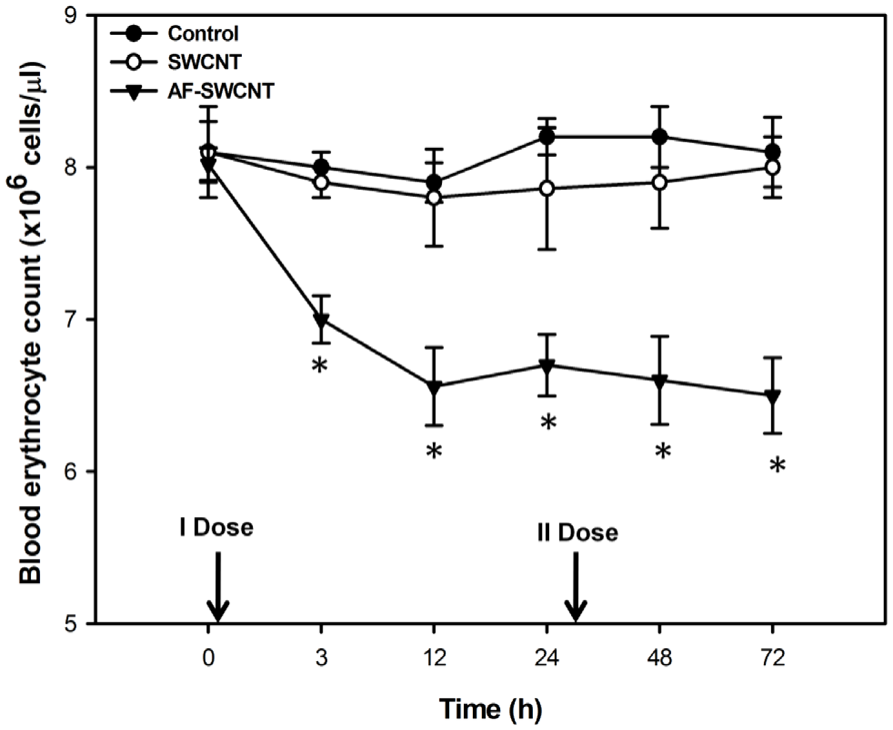
Prolongation of anemia by a second dose of AF-SWCNTs. The experiment was performed as detailed in Figure 5 except that a second dose of SWCNTs and AF-SWCNTs was given 25 h after the first dose. Blood erythrocyte counts were monitored till three days. All data points represent mean ± SEM of three observations. *p<0.01 as compared to PBS administered control groups.

Results above show that treatment with AF-SWCNTs resulted in a transient anemia in mice. A second dose of AF-SWCNTs given 24 h after the first dose resulted in a more sustained anemia that was not alleviated even at 72 h time point (Figure 7). Administration of two doses of control SWCNTs however still had no significant effect on the blood erythrocyte count.

### Age dependence of susceptibility of blood erythrocytes to AF-SWCNTs

**Figure 8:**
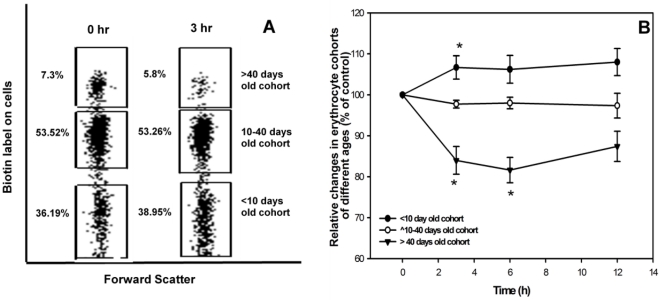
Effect of single intravenous dose of AF-SWCNTs on the proportions of erythrocyte cohorts of different age groups in mouse blood. Mice were prepared by DIB technique as described in materials and methods. AF-SWCNTs (100 µg) was administered i.v. and proportions of erythrocyte cohorts of different ages in blood circulation were monitored. Erythrocyte cohorts were identified by biotin levels on erythrocytes as per the DIB technique [9]. Results of a representative experiment in panel A show the distribution of the three erythrocytes cohorts [Old erythrocytes, (biotin^high^, age>40 days, top boxes); intermediate age erythrocytes(biotin^low^ age10–40 days, middle boxes); young erythrocytes (biotin^negative^ age<10 days, lower boxes)] before and 3h after intravenous administration of AF-SWCNT (100 µg) in 6–8 weeks old Swiss mice. Blood samples were also taken from these mice at different intervals of time and effect of AF-SWCNT administration on the distribution of the three erythrocyte cohorts was assessed at each time points (panel B). Proportion of young, intermediate and old erythrocytes at zero time point were taken as hundred and changes induced by AF-SWCNTs have been depicted as percent of control. Each data point in panel B represents mean ± SEM of data from swiss mice. *p<0.05 as compared to control groups by ANOVA.

Results so far indicate that the intravenous administration of AF-SWCNTs caused a marked and transient anemia in mice. It was of interest to determine if the toxic effect of AF-SWCNTs was generalize or was selective for erythrocytes of specific age groups. To determine the susceptibility of erythrocytes of different age groups, we used a technique that we recently developed to enumerate circulating erythrocyte cohorts of different age groups [9]. In this technique that involves a two step *in vivo* biotinylation of circulating erythrocytes, erythrocytes of different age groups can be identified as a biotin negative population (young erythrocytes, age <10 days), a biotin high population (Old erythrocytes, age>40 days) and a biotin low population (erythrocytes of age between 10 to 40 days). Results in Figure 8A show that just 3 h after a single dose of AF-SWCNTs, the proportion of old erythrocyte (age>40 days) in blood fell from 7.3% to 5.8%, whereas the proportion of young erythrocytes (age<10days) increased from 36.19% to 38.95%. No significant change occurred in the erythrocytes of intermediate age group (age 10–40 days). Time kinetics of changes in the proportion of old and young erythrocytes is shown in Figure 8B. These results suggest that the older erythrocytes in blood circulation may be most susceptible to the toxic effect of AF-SWCNTs.

## Discussion

Our first set of experiments indicated that the AF-SWCNTs had a marked toxic effect on erythrocytes *in vitro*. The effect was dose and time dependent and after one day of incubation with AF-SWCNT, 70–90% of the erythrocytes were lost at all the doses (10, 25 and 50 µg/ml) of AF-SWCNT. It was also interesting to observe that the damaging effect is seen earlier with the highest dose of 50 µg/ml leading to 70–90% decline in recovery after just 10–12 h while interaction ensures the similar damaging effect on erythrocytes treated with lower doses also (10 and 25 µg/ml) when kept for a longer duration (24 h). It should be noted that the process to generate AF-SWCNTs involved extensive dialysis for several days and it is therefore unlikely that some material leaching from AF-SWCNTs was responsible for the toxic effect on erythrocytes. To further test this possibility, we separated AF-SWCNTs from erythrocytes by a dialysis membrane (10,000 mol wt cut off) during incubation and found that no loss of erythrocytes occurred (results not shown). Interestingly control SWCNTs had no significant effect on survival of erythrocytes. The reason for this lack of effect of control SWCNTs could be that the control SWCNTs preparations are highly agglomerated and do not interact effectively with cells. These results corroborate our previous findings on the effects of control and acid functionalized SWCNTs on LA4 lung epithelial cells lines where too the AF-SWCNTs induced a marked cellular damage but control SWCNTs lacks a significant cytopathic effect [5].

Using fluorescence tagged AF-SWCNTs, we could demonstrate a significant binding of AF-SWCNTs with erythrocytes. Flow cytometric analysis indicated that almost 70% of erythrocytes incubated with fluorescence tagged AF-SWCNTs bound nanotubes. Further confocal microscopic studies suggested that some AF-SWCNTs could enter the erythrocyte also. Since an active uptake of AF-SWCNTs by erythrocytes appear unlikely, the observed association of AF-SWCNTs could reflect loose binding of AF-SWCNTs with erythrocyte membrane or a stronger binding perhaps reflecting embedding of nanotubes in erythrocyte membrane or even some passive entry of the nanotubes into the cells. Our results showing that 18% of the erythrocytes retained the association with fluorescence tagged AF-SWCNTs even after three rigorous washings suggest that AF-SWCNTs may interact effectively with erythrocytes.

Membrane damage as well as PS externalization seen in response to treatment with AF-SWCNTs could be a direct consequence of interactions of the AF-SWCNTs with erythrocytes. Erythrocytes treated with AF-SWCNTs were found to have a marked increase in ANS binding within one hour of treatment indicating that the membrane damage in erythrocytes may be early consequence of interaction with AF-SWCNTs. Erythrocytes treated with AF-SWCNTs also displayed increasing externalization of PS in the membrane that was related to the dose and time period of treatment. While these results clearly show a response similar to apoptotic response in nucleated cells, it is not clear from our results if PS externalization alone lead to the death/removal of erythrocytes. We also examined the AF-SWCNT induced erythrolysis by time lapse micrography (Video S1) and found that the AF-SWCNT exposed erythrocyte gradually disintegrated and disappeared from the field in a random manner and this process started within 30 min of treatment with 75 µg/ml of AF-SWCNTs.

Results of *in vitro* studies established the toxic effect of AF-SWCNTs on erythrocytes, which was further confirmed by *in vivo* studies. Intratracheal and intravenous administration of AF-SWCNTs induced transient anemia in mice. As observed *in vitro*, control SWCNT preparations did not result in decrease in erythrocyte count. Interestingly, intra-peritoneal and intra-gastric administration of AF-SWCNTs didn't reduce the RBC count too. The drop in erythrocyte count in blood seen in case of intravenous administration of AF-SWCNTs could be due to an efficient interaction of the nanotubes with erythrocytes in circulation. In all other forms of in *vivo* treatments, nanotubes were not deposited into blood circulation directly. As a result, the immediate physical contact of nanotubes with erythrocytes that was possible in case of intravenous administration only, would not have taken place. Significant anemia in response to intratarcheal administration of AF-SWCNTs could be a result of the relatively easy diffusion of nanotubes through thin alveolar membranes as reported earlier also [16]. Diffusion of CNTs from peritoneal cavity and stomach into bloodstream may be slow and could be the reason for the lack of effect of AF-SWCNTs in these cases. In another recent study, SWCNTs were not found to induce significant toxic effects in nude mice [17]. The processes of preparation of stable suspensions of SWCNTs in this study were however very different from the one used in our study and these differences could be responsible of the results in these two studies.

Acute anemia is known to stimulate the release of erythrocytes from spleen and bone marrow in order to compensate for the loss of erythrocytes [18], [19]. Recovery of erythrocytes count in blood may represent a homeostatic response to fall in blood erythrocyte count. This propositions is supported by our observation that a significant increase in blood reticulocyte count was observed in blood with few hours of i.v. infusion of AF-SWCNTs (results not shown). A second dose of AF-SWCNTs given intravenously extended the duration of anemia. The fact that the anemia induced by intravenous administration of AF-SWCNTs was transient in nature suggests that the nanotubes may have a short half life in circulation. In a recent study, radio-labelled polydispersed SWCNTs were shown to accumulate in mouse liver [20]. It is therefore possible that the nanotubes may be removed from circulation by organs like liver and kidney.

There could be several possible mechanisms of induction of anemia in response to AF-SWCNTs. A direct cytotoxic effect of nanotubes on erythrocytes is suggested by our *in vitro* experiments. It was important to understand whether the cytotoxic effect of nanotubes was exerted uniformly on all erythrocytes in circulation or it was related to the age of erythrocytes in circulation. This question could be addressed by help of a technique involving two steps of *in vivo* biotinylation of circulating erythrocytes that has recently been developed in our laboratory. By using this technique it became clear that the oldest subpopulation of erythrocytes (age > 40 days) was specifically sensitive to nanotubes. Blood count of young erythrocytes (<10 days) actually increased after administration of AF-SWCNTs. This increase could be a consequence of release of fresh erythrocytes into circulation from spleen and bonemarrow. Interestingly the erythrocytes population of intermediate age group (10–40days) didn't change in response to AF-SWCNTs. These results suggest that the older erythrocytes in circulation that have become weakened by oxidative damage may be easier targets for the nanotubes. It should however be stressed that even though greater susceptibility of older erythrocytes to AF-SWCNTs is indicated by the *in vivo* experiments, a more generalized erythrolysis observed *in vitro* (Figure 1) suggests that age related differences in susceptibility observed *in vivo* may only be relative and not absolute.

In conclusion, we have shown an acute cytotoxic effect of polydispersed SWCNTs *in vitro* as well as *in vivo*. Rapid induction of anemia within a day is followed by a recovery of blood count of erythrocytes. Effect of AF-SWCNTs on the erythropoietic activity in spleen and bone marrow will further clarify if the fall in blood count of erythrocytes is accompanied with changes in erythropoietic activity. These studies using fluorescence tagged AF-SWCNTs are currently under way in our laboratory.

## Supporting Information

Video S1 Time lapse recording demonstrating the lysis of erythrocytes incubated with AF-SWCNTs. Erythrocytes from Swiss mice were suspended in RPMI+1%FBS with 75 µg/ml AF-SWCNTs in tissue culture dish (35 mm) and were placed on the observation platform of a live cell imaging (Nikon Eclipse Ti) microscope in a chamber maintained at 37C in 5% CO2 atmosphere. Cells were allowed to settle for 30 min before recording was started.
(AVI)
